# Macrophage transition to a myofibroblast state drives fibrotic disease in uropathogenic *E*. *coli*-induced epididymo-orchitis

**DOI:** 10.1172/JCI193793

**Published:** 2025-10-01

**Authors:** Ming Wang, Xu Chu, Zhongyu Fan, Lin Chen, Huafei Wang, Peng Wang, Zihao Wang, Yiming Zhang, Yihao Du, Sudhanshu Bhushan, Zhengguo Zhang

**Affiliations:** 1 Medical Research Center, The First Affiliated Hospital of Zhengzhou University, Zhengzhou, Henan, China.; 2 The First Affiliated Hospital of Henan University of Science and Technology, Luo Yang, Henan, China.; 3 Department of Urology, and; 4 Department of Biological Sample Bank, The First Affiliated Hospital of Zhengzhou University, Zhengzhou, Henan, China.; 5 Department of Urology, Daping Hospital, Army Medical University, Chongqing, China.; 6 Institute of Anatomy and Cell Biology, Unit of Reproductive Biology, Justus-Liebig-University Giessen, Giessen, Germany.

**Keywords:** Infectious disease, Inflammation, Bacterial infections, Fibrosis, Macrophages

## Abstract

Bacterial infections, particularly uropathogenic *E*. *coli* (UPEC), contribute substantially to male infertility through tissue damage and subsequent fibrosis in the testis and epididymis. The role of testicular macrophages (TMs), a diverse cell population integral to tissue maintenance and immune balance, in fibrosis is not fully understood. Here, we used single-cell RNA sequencing in a murine model of epididymo-orchitis to analyze TM dynamics during UPEC infection. Our study identified a marked increase in S100a4^+^ macrophages, originating from monocytes, strongly associated with fibrotic changes. This association was validated in human testicular and epididymal samples. We further demonstrated that S100a4^+^ macrophages transition to a myofibroblast-like phenotype, producing extracellular matrix proteins such as collagen I and fibronectin. S100a4, both extracellular and intracellular, activated collagen synthesis through the TGF-β/STAT3 signaling pathway, highlighting this pathway as a therapeutic target. Inhibition of S100a4 with niclosamide or macrophage-specific *S100a4* KO markedly reduced immune infiltration, tissue damage, and fibrosis in infected murine models. Our findings establish the critical role of S100a4^+^ macrophages in fibrosis during UPEC-induced epididymo-orchitis and propose them as potential targets for antifibrotic therapy development.

## Abstract 2

The accumulation of fibroblast-like features of S100a4+ testicular macrophages promotes tissue fibrosis during UPEC infection.

## Introduction

Uropathogenic *E*. *coli* (UPEC) is a leading cause of epididymitis/orchitis and often results in substantial tissue damage and fibrosis (1, 2), a leading but potentially reversible cause of male infertility (3, 4). As an immune privileged organ, testicular leukocytes have attenuated capacity to combat foreign antigens (5), allowing limited immune responses to protect spermatogenesis (6, 7). However, certain bacterial infections can overcome this inhibition to trigger inflammation and injury of the testes. Antibiotics have been the mainstay therapy for epididymo-orchitis, but inflammatory damage and fibrosis often persist even after pathogen has been eradicated. This residual fibrosis and tissue damage can disrupt sperm production and transport, potentially resulting in male infertility (8, 9).

Macrophages play critical roles in host defense and tissue homeostasis across diverse body sites, where they adopt distinct phenotypes and functions both in the steady state and during inflammation. Recent studies suggest that testicular macrophages (TMs) comprise multiple subtypes with specialized roles (10), but it is unclear which of these populations contribute to fibrosis in the context of bacterial infection. In animal models, LPS exposure or UPEC infection can stimulate influx of peripheral monocytes and promote tissue damage and fibrosis (1, 11). Conversely, blocking infiltration of Ly6C^hi^ monocytes substantially reduced murine testicular damage and fibrosis in an *E*. *coli*–induced orchitis model (12). Our previous study further indicated that testes contain 2 different macrophage populations with distinct origins and divergent functions: interstitial macrophages and peritubular macrophages (1). However, it remains unknown how these TM subtypes contribute to postinfection testicular fibrosis.

Testicular/epididymal fibrosis results from dysregulated tissue repair in the context of inflammation, leading to excessive deposition of extracellular matrix (ECM) (13, 14). Fibroblast activation is considered a critical part of this process, yet evidence of a role for these cells in the testicular interstitium is currently lacking (15). Macrophage-to-myofibroblast transition (MMT) is an alternative pathway of tissue fibrosis that can be activated under inflammatory conditions (16, 17), but it is unknown whether this mechanism contributes to testicular/epididymal fibrosis triggered by UPEC infection. It is well established that blocking monocyte recruitment by CCR2 KO or clodronate-mediated depletion notably reduces tissue destruction and fibrosis in experimental orchitis models (1, 12, 13), but the TM subgroups that regulate these events during infection-induced inflammation remain poorly defined.

In this study, we explored mechanisms by which TM subtypes regulate infection-induced inflammation and tissue fibrosis, as well as the impact of targeted therapy. To do this, we constructed a UPEC-induced epididymitis/orchitis model and identified key cellular and molecular mediators of post–inflammatory fibrosis, using a combination of in vivo and in vitro experiments, including single-cell RNA-Seq (scRNA-Seq). Our findings shed important light on potential targets for antifibrotic therapy in epididymitis/orchitis.

## Results

### UPEC infection remodels the murine TM compartment.

To obtain a comprehensive overview of the testicular immune compartment in both steady state and infectious settings, we FACS-sorted live CD45^+^ leukocytes from UPEC-infected and sham-operated control testes on days 7 and 14, then used the 10x Genomics Chromium platform to perform scRNA-Seq of 31,564 isolated cells (Supplemental Figure 1A; supplemental material available online with this article; https://doi.org/10.1172/JCI193793DS1). We next performed *t*-distributed stochastic neighbor embedding unbiased cluster analysis, which identified multiple populations of testicular immune cells, including macrophages (*C1qa*, *C1qc*), T cells (*Cd163l1*, *Trdc*), B cells (*Lgkc*, *Ebf1*), monocytes (*Plc8*, *Gda*), granulocytes (*S100a8*/*S100a9*), and NK cells (*Xcl1*, *Gm2682*) (Supplemental Figure 1, B–D, and Supplemental Figure 2, A and B). Among these, macrophages and T cells were the most abundant cell types with a rich set of subclusters in the testis (Supplemental Figure 2C). After UPEC infection, the numbers and percentages of immune cells increased by day 7 and day 14, with multiple lineages exhibiting subclusters with distinct gene expression profiles at each time point. In particular, TMs displayed increased heterogeneity and expression of granulocyte and monocyte marker genes after UPEC infection, especially at the 7-day time point (Supplemental Figure 1, C and D, and Supplemental Figure 2C), suggesting potential remodeling of this compartment over the course of inflammation.

**Figure 1:**
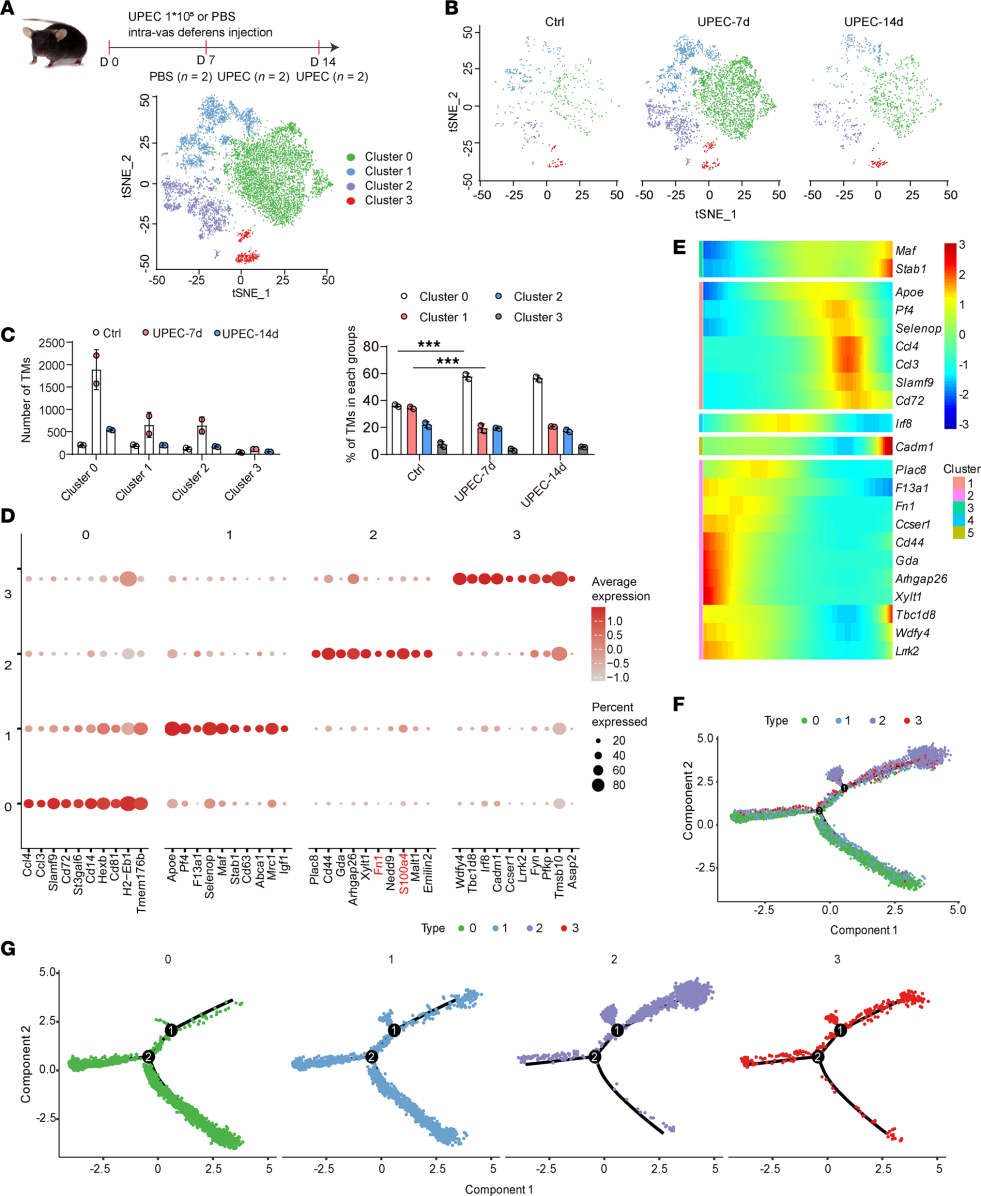
UPEC infection modifies TM subtype distribution. (**A** and **B**) Schematic representation of the UPEC-induced epididymo-orchitis mouse model *t*-distributed stochastic neighbor embedding (*t*-SNE) analysis of TM subpopulations in the testes after UPEC infection, shown in summary and by different groups. (**C**) Bar graphs displaying change in numbers and proportions of TMs at different time points after UPEC infection. Data are presented as the mean ± SD. ***P < 0.001, by 1-way ANOVA followed by Tukey’s multiple-comparison test. (**D**) Dot plot showing differential gene expression across clusters. (**E**–**G**) Heat map of top 22 differentially expressed genes along with pseudotime analysis (**E**). Monocle prediction of macrophage developmental trajectory (**F**). Monocle prediction of TM developmental trajectory, with each Seurat-based cluster shown separately (**G**). Ctrl, control.

To better understand the effects of UPEC infection on TM subtypes, we conducted further analysis of the major populations identified by scRNA-Seq. Based on gene expression characteristics, postinfection TMs were categorized into 4 main subgroups: cluster 0 (*Ccl4*, *Ccl3*), cluster 1 (*Apoe*, *Pf4*), cluster 2 (*Cd44*, *Plac8*), and cluster 3 (*Wdfy4*, *Tbc1d8*) (Figure 1, A–D, and Supplemental Figure 3, A and B). Quantitative analysis of each cluster revealed distinct patterns: All Clusters were minimally represented in the control group, significantly increased on postinfection day 7, then markedly decreased by day 14. Conversely, clusters 0 and 1 exhibited divergent trends at 7 and 14 days after infection (Figure 1C). Interestingly, we identified that cluster 2 macrophages highly expressed both fibronectin (*Fn*) and *S100a4*, also known as fibroblast-specific protein (*Fsp*), which are thought to play key roles in promoting tissue fibrosis (Figure 1D and Supplemental Figure 3C). Gene Ontology enrichment analysis also indicated that multiple fibrosis-related pathways were enriched in cluster 2 macrophages, including collagen trimer, integrin binding, and s100 protein binding (Supplemental Figure 4).

To assess potential relationships between TM subclusters, we next analyzed the macrophage compartment using the Monocle algorithm. We observed that cluster 2 macrophages localized at the beginning of the pseudotime trajectory, whereas cluster 0 and Cluster 1 macrophages were situated at the end of the trajectory. Cluster 3, which displayed a similar pseudotime trajectory to cluster 2, was localized at the initiating branch (Figure 1, E–G, and Supplemental Figure 3, D and E). Tracking change in gene expression across macrophage clusters revealed developmental patterns that defined the phenotype and function of each TM subtype. Cluster 2 macrophages were characterized by high expression of *Plac8* and *Cd44*, whereas cluster 3 was defined by *Wdfy4* and *Tbc1d8*, consistent with these being monocyte-derived cells recruited during tissue inflammation, as also identified in our previous study (1). In contrast, clusters 0 and 1 displayed features of TM cells, including high expression of *Slamf9*, *Cd72*, and *Mrc1*, indicating that these correspond to tissue-resident populations. Together, these data indicate UPEC infection conspicuously remodels the TM compartment during different stages of inflammation.

### S100a4^+^ macrophages accumulate in testes after UPEC infection.

**Figure 2:**
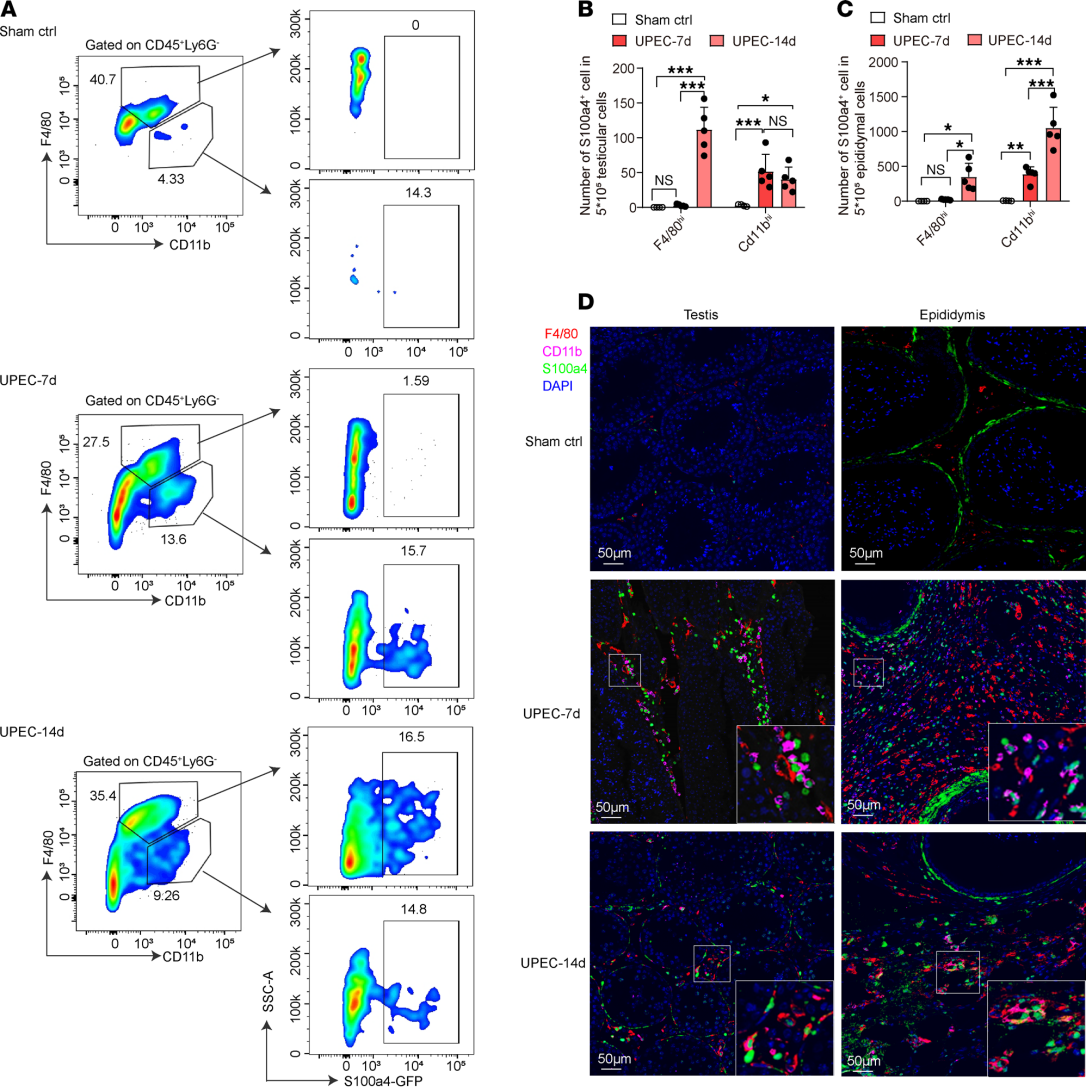
UPEC infection promotes accumulation of S100a4^+^ TMs in the testes and epididymis. (**A**–**C**) A UPEC-induced epididymo-orchitis model was developed in S100a4-GFP transgenic mice. Flow cytometry identified the percentages and absolute numbers of GFP^+^ cells within the F4/80^+^CD11b^+^ TM population at postinfection days 7 and 14. Representative gating strategy images in testes are presented (**A**). Data are presented as the mean ± SD. *n* = 5. *P < 0.05, **P < 0.01, and ***P < 0.001, by 1-way ANOVA with Tukey’s multiple-comparison test. (**D**) IF staining assessed the expression and colocalization of S100a4 (green), F4/80 (red), and CD11b (pink) in the testes and epididymis on day 7 after UPEC infection. Nuclei were counterstained with DAPI (blue). Scale bar: 50 μm. Ctrl, control.

S100a4 has been reported to play important roles in multiple fibrotic settings, but it is unknown how this protein contributes to testicular fibrosis after UPEC infection. To verify the accumulation of S100a4^+^ TMs after UPEC exposure, we investigated the tissue dynamics of this population using S100a4-GFP transgenic mice (expressing EGFP under the control of the S100a4 promoter). In the UPEC-induced epididymo-orchitis model, we observed that S100a4-GFP^+^F4/80^+^ TMs were significantly increased in the testes and epididymis at day 7 after infection compared with the sham surgery control group (Figure 2, A–C). Further analysis indicated that the S100a4-GFP^+^ cells were primarily F4/80^lo^CD11b^hi^ cells, suggesting that they mainly originated from monocytes. Also, S100a4-GFP^+^ bone marrow cell transplantation (BMT) experiments demonstrated that CD11b^hi^S100a4^+^ cells in the testes and in the epididymis displayed notable S100a4-GFP expression (Supplemental Figure 5, A–C). Moreover, scRNA-Seq data confirmed that cluster 2 TMs had high *Ccr2* expression (Supplemental Figure 5D). Collectively, these findings provide conclusive evidence that S100a4^+^ TMs are monocyte derived. However, S100a4 was also expressed by F4/80^hi^ macrophages at day 14 after infection, suggesting local differentiation of S100a4^+^ monocytes into tissue macrophages in both the testis and epididymis (Figure 2, A–C). This finding was further supported by immunofluorescence (IF) staining, which indicated that UPEC infection led to an increase in both F4/80^+^ and S100a4^+^CD11b^+^ cell populations within the testes and epididymis (Figure 2D). Importantly, because S100a4 is also known to be expressed in fibroblasts (18), we confirmed that S100a4^+^ cells were clearly present in the smooth muscle layer of the epididymis.

### S100a4^+^ TMs promote fibrosis in the testes and epididymis.

**Figure 3:**
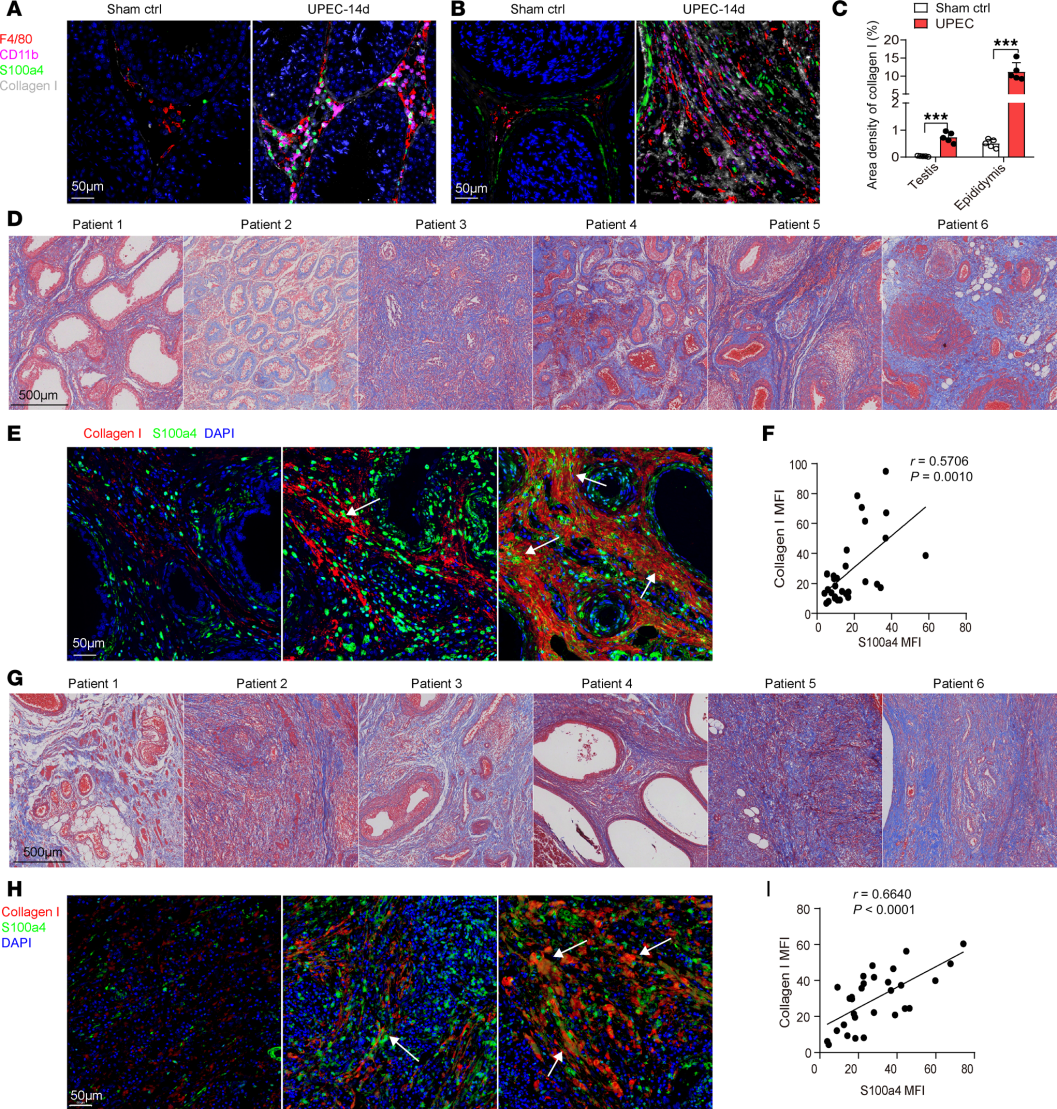
UPEC infection-induced accumulation of S100a4^+^ cells and collagen I in the testes and epididymis. (**A** and **B**) IF staining was used to assess expression and localization of F4/80 (red), CD11b (pink), S100a4 (green), and collagen I (gray) within the testes (**A**) and epididymis (**B**) at day 14 after UPEC infection. Nuclei were counterstained with DAPI (blue). Scale bar: 50 μm. (**C**) Bar blot summarizing the area of collagen I density in testis and epididymis. *n* = 5. ***P < 0.001, by Student’s *t* test. (**D** and **G**) Masson staining demonstrating varying degrees of fibrosis within testicular and epididymal tissue from different patients. (**E** and **H**) Expression and colocalization of S100a4 (green) and collagen I (red) were visualized by IF staining across diverse regions of the testis and epididymis. (**F** and **I**) Image J software facilitated analysis and MFI quantification of S100a4 and collagen I across tissue regions. Pearson’s correlation analysis indicated a positive relationship between S100a4 MFI and collagen I MFI (sample size *n* = 30; *n* = 5 regions per sample). The resulting Spearman’s correlation coefficient (*R*) and corresponding *P* value are reported. Ctrl, control.

To ascertain the role of S100a4^+^ TMs in fibrosis, we next examined the frequency of this population and their colocalization with collagen I in the testes and epididymis at day 14 after UPEC infection. Multiplex IF staining revealed a significant increase in both the number of F4/80^+^CD11b^+^S100a4^+^ cells and in collagen I levels within the testes and epididymis after infection (Figure 3, A–C). Importantly, similar patterns were observed in human testicular samples obtained from surgical procedures, including Masson staining to confirm the presence of pathological fibrosis (Figure 3D). Further IF indicated extensive colocalization of S100a4 with collagen I (Figure 3E) and significant positive correlation of S100a4 fluorescence density with collagen I content (Figure 3F). Similar colocalization and positive correlation of S100a4 with collagen I also were noted in fibrotic areas of human epididymal samples (Figure 3, G–I). These findings strongly suggest S100a4^+^ macrophages are involved in the development of epididymal/testicular fibrosis in mouse models and human tissues.

### Fibroblast-like features of S100a4^+^ TMs.

**Figure 4:**
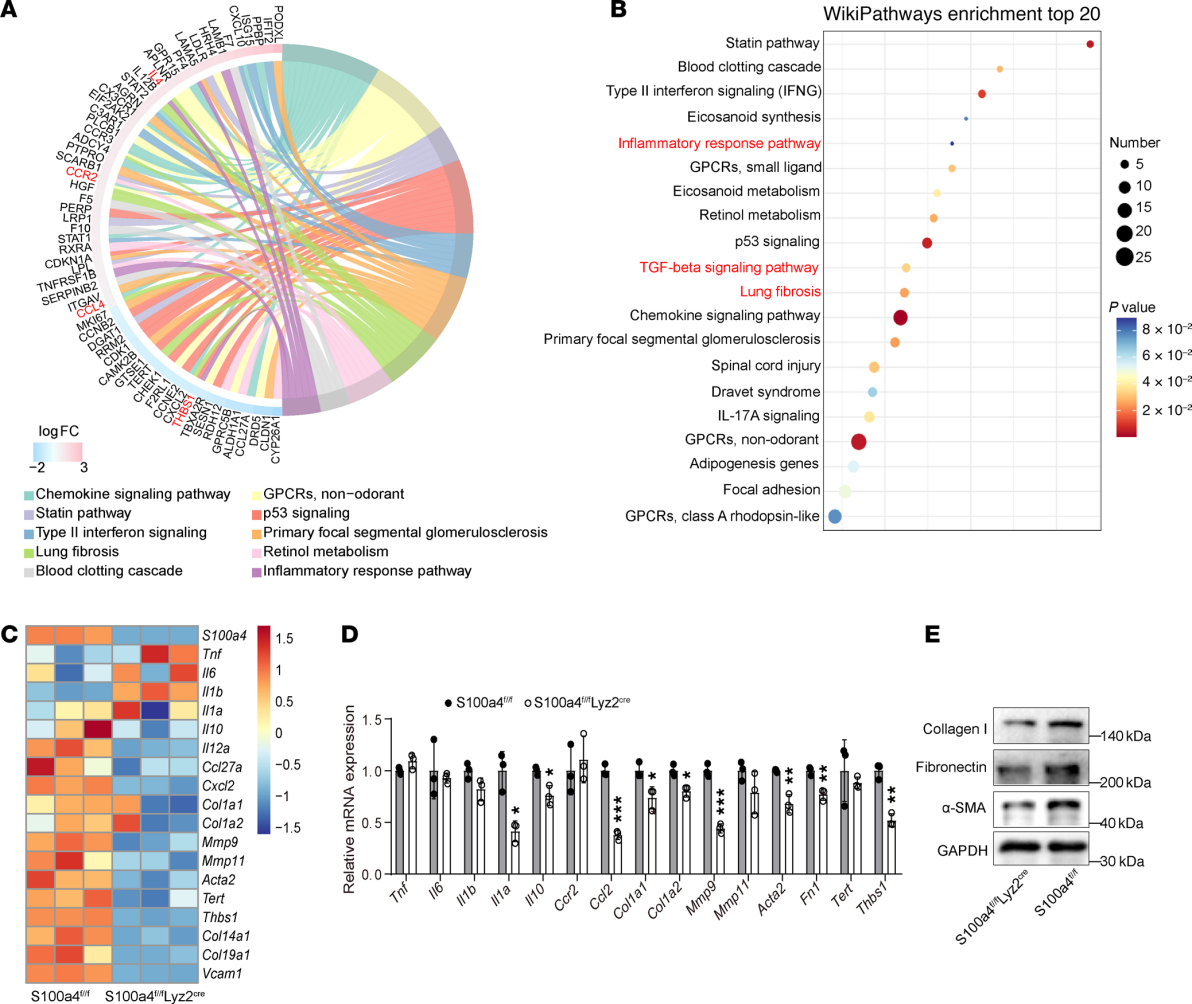
S100a4^+^ macrophages express ECM proteins. (**A** and **B**) BMDMs were generated from the BM of S100a4^f/f^ or S100a4^f/f^ Lyz2^cre^ mice via culture for 7 days with M-CSF (20 ng/mL). Gene expression was detected using RNA-Seq analysis. WikiPathways enrichment analysis identified the differentially expressed genes (**A**) and top 20 enriched pathways (**B**). (**C**) Selected inflammatory and fibrosis-related genes in the 2 groups. (**D** and **E**) qRT-PCR and Western blot validation of differentially expressed genes and proteins in *S100a4^fl/fl^* and *S100a4^fl/fl^* *Lyz2^Cre^* BMDMs. Data are presented as the mean ± SD. *n* = 3. *P < 0.05, **P < 0.01, and ***P < 0.001, by Student’s *t* test.

The results reported in the preceding section indicated S100a4^+^ macrophage frequency was positively correlated with fibrosis in both the testis and epididymis. To investigate the underlying mechanism, we performed RNA-Seq to identify differentially expressed genes in bone marrow–derived macrophages (BMDMs) isolated from macrophage-specific S100a4 KO mice (S100a4^f/f^Lyz2^cre^). WikiPathways enrichment analysis indicated substantial enrichment of multiple fibrosis-related pathways, including the lung fibrosis and TGF-β signaling pathways, among the differentially expressed genes in S100a4-expressing macrophages (Figure 4, A and B). Moreover, heat map and qRT-PCR results confirmed that expression of fibrosis-associated genes such as *Col1a1*, *Col1a2*, *Mmp9*, *Mmp11*, *Cxcl2*, *Acta2*, and *Thbs1* were significantly higher in S100a4-expressing BMDMs (Figure 4, C and D), whereas expression of proinflammatory genes did not substantially differ. Western blot analysis also revealed increased levels of ECM proteins in S100a4^+^ macrophages, specifically collagen I, fibronectin, and fibroblast activation marker α-SMA (Figure 4E).

### S100a4 promotes a myofibroblast state in BMDMs via TGF-STAT3 signaling.

**Figure 5:**
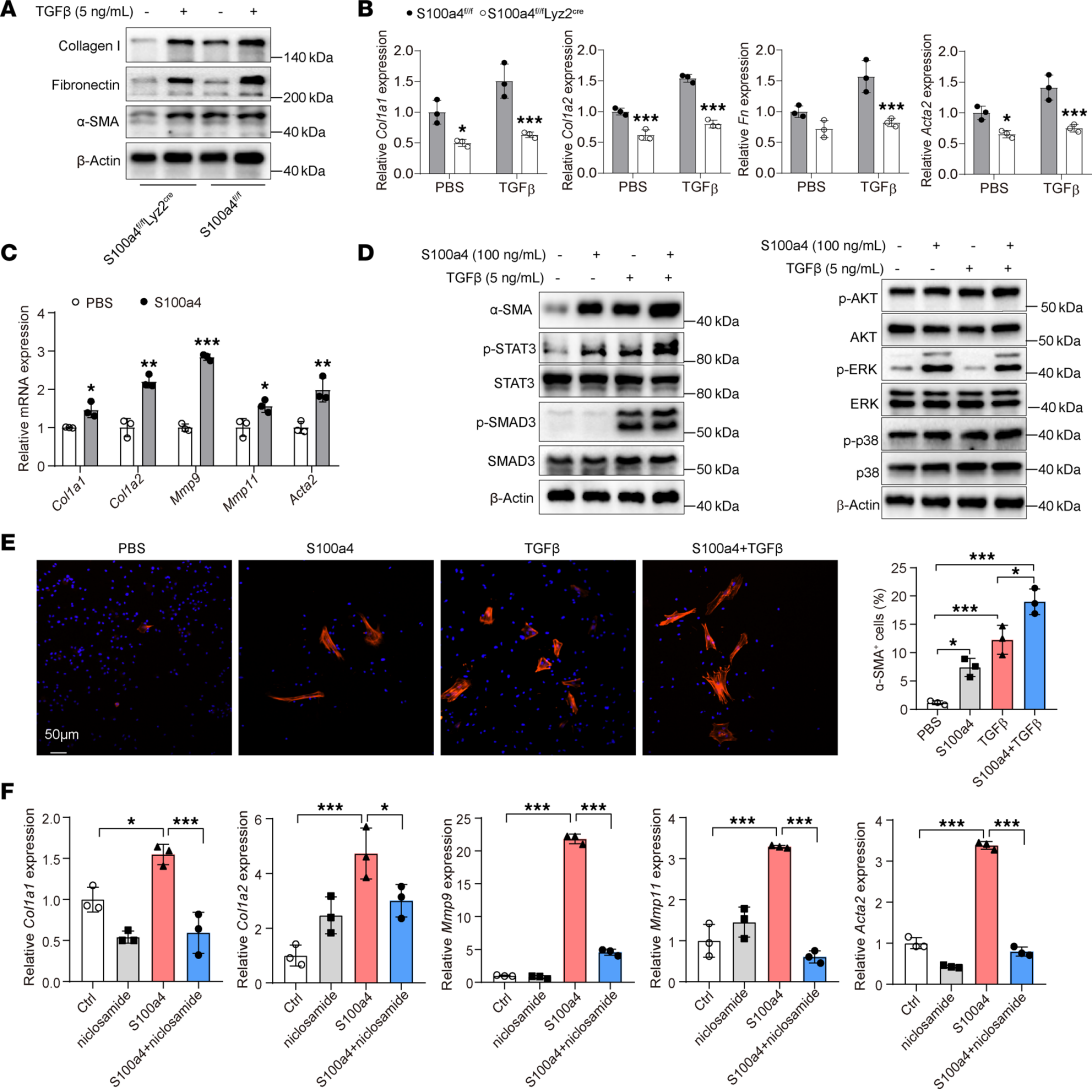
S100a4 activates the p-STAT3 signaling pathway in macrophages. (**A** and **B**) BMDMs were generated from *S100a4^fl/fl^ Lyz2^Cre^* or *S100a4^fl/fl^* mice and then treated with TGF-β (5 ng/mL) prior to analysis of collagen I, fibronectin, and α-SMA gene/protein expression. (**C**) BMDMs were treated with S100a4 (100 ng/mL), and mRNA was detected using qRT-PCR. (**D** and **E**) BMDMs were treated with either TGF-β (5 ng/mL) or S100a4 (100 ng/mL) and then probed for expression of α-SMA, p-STAT3, SMAD, AKT, ERK, and p38-related proteins by Western blotting (**D**) or IF (**E**). Scale bar: 50 μm. Representative images are shown, and a bar plot summarizes the percentage of α-SMA^+^ cells (red) among BMDMs. (**F**) The BMDMs were treated with either S100a4 (100 ng/mL) or niclosamide (10 μM), or a combination of both. Relative mRNA levels were determined using qRT-PCR. Data are presented as the mean ± SD. *n* = 3. *P < 0.05, **P < 0.01, and ***P < 0.001, by Student’s *t* test (**B** and **C**) or 1-way ANOVA followed by Tukey’s multiple-comparison test (**E** and **F**). Ctrl, control.

The TGFβ-SMAD-STAT3 signaling pathway plays crucial roles in fibrosis (19, 20), and previous data indicate S100a4 can amplify TGF-β–induced fibroblast activation (21). Therefore, we proceeded to test ECM protein expression in BMDMs upon TGF-β stimulation. We observed that TGF-β upregulates expression of collagen I, fibronectin, and α-SMA proteins in BMDMs, especially in S100a4-expressing macrophages (Figure 5A), which was also confirmed at the mRNA level (Figure 5B).

Considering the secretory nature of S100a4, we next treated BMDMs with S100a4 recombinant protein. Treatment with S100a4 significantly upregulated mRNA levels of *Col1a1*, *Col1a2*, *Mmp9*, *Mmp11*, and *Acta2* as well as expression of a-SMA (Figure 5, C and D). Moreover, S100a4 treatment activated STAT3, thereby enhancing the TGFβ-STAT3 signaling pathway, while having no effect on SMAD3, AKT, or p38 signaling pathways. Additionally, S100a4 alone could stimulate the ERK-associated pathway, which is involved in fibroblast activation and proliferation (Figure 5D). IF staining confirmed that S100a4 alone, or in combination with TGF-β, induced α-SMA expression in BMDMs (Figure 5E).

Consistent with earlier reports that S100a4 exerts proinflammatory effects via STAT3 activation in liver cells (22), our results indicated that S100a4 treatment also increased phosphorylated STAT3 (p-STAT3) levels in BMDMs. Accordingly, the S100a4 inhibitor niclosamide reversed the S100a4-induced expression of *Col1a1*, *Col1a2*, *Mmp9*, *Mmp11*, and *Acta2* in BMDMs (Figure 5F). It was previously demonstrated that expression of α-SMA in inflammatory macrophages is associated with the MMT state (23). Our results suggest, therefore, that S100a4 autocrine signaling may drive BMDMs toward a myofibroblast phenotype that exerts profibrotic activity.

### Macrophage S100a4 deletion inhibits UPEC-induced fibrosis of the epididymis/testis.

**Figure 6:**
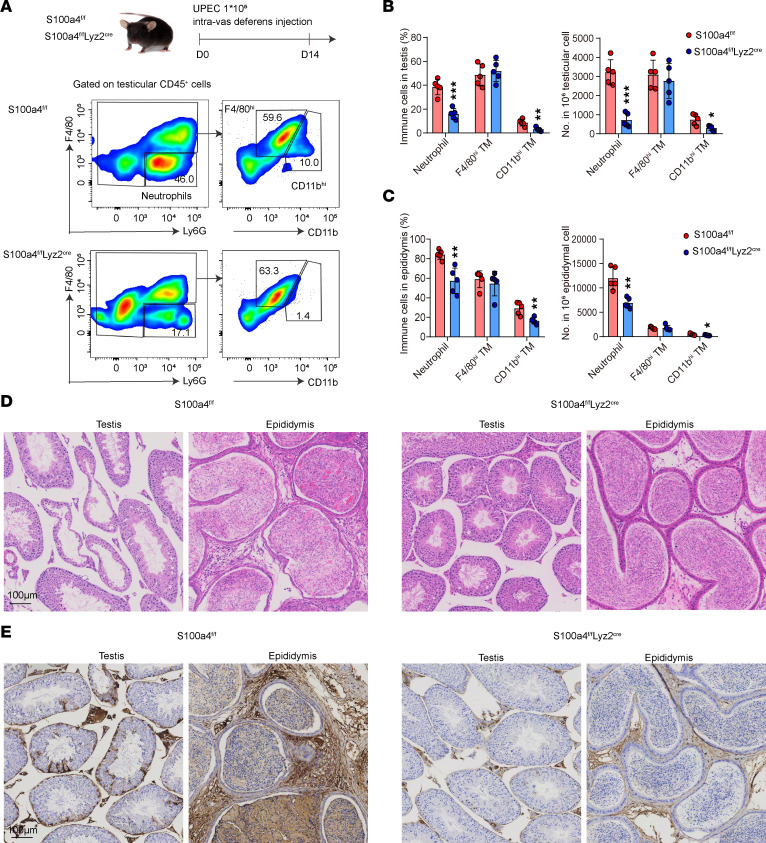
Macrophage S100a4 deletion inhibits fibrosis in epididymis/testis after UPEC infection. (**A**–**C**) The UPEC-induced epididymo-orchitis model was established in S100a4^f/f^ Lyz2^cre^ and S100a4^f/f^ mice. Flow cytometry analysis assessed the quantity and proportions of immune cells in testis and epididymis at day 14, including frequencies of CD45^+^F4/80^–^Ly6G^+^ neutrophils, CD45^+^F4/80^hi^ TMs, and CD45^+^CD11b^hi^ TMs. A representative image of the testis is shown (**A**); bar plots summarize all samples with cell percentage (left) and number (right) in the testis (**B**) and epididymis (**C**). *n* = 5. Data are presented as the mean ± SD. *P < 0.05, **P < 0.01, and ***P < 0.001, by Student’s *t* test. (**D**) H&E staining was used to analyze the structure of testicular and epididymal tissues. (**E**) Immunohistochemical staining of collagen I levels in the testis/epididymis. Scale bar: 100 μm.

To further validate our finding that S100a4^+^ TMs mediate testicular and epididymal fibrosis after UPEC infection, we next established a UPEC-induced epididymo-orchitis model in myeloid cell–specific S100a4-KO mice (*S100a4^fl/fl^ Lyz2^Cre^*). Flow cytometric analysis conducted 14 days after infection revealed a marked reduction in numbers and percentages of neutrophils and monocyte-derived CD11b^hi^ TMs in the testes of *S100a4^fl/fl^ Lyz2^Cre^* mice (Figure 6, A–C). In line with these findings, H&E staining as well as immunohistochemical analysis confirmed that tissue damage and fibrosis in the testes and epididymis were substantially reduced in *S100a4^fl/fl^ Lyz2^Cre^* mice relative to *S100a4^fl/fl^* control mice (Figure 6, D and E). These data strongly indicate S100a4^+^ macrophages newly recruited in response to UPEC infection are a critical population driving testicular and epididymal inflammation and fibrosis in vivo.

### Niclosamide inhibits fibrosis in the testes and epididymis after UPEC infection.

**Figure 7:**
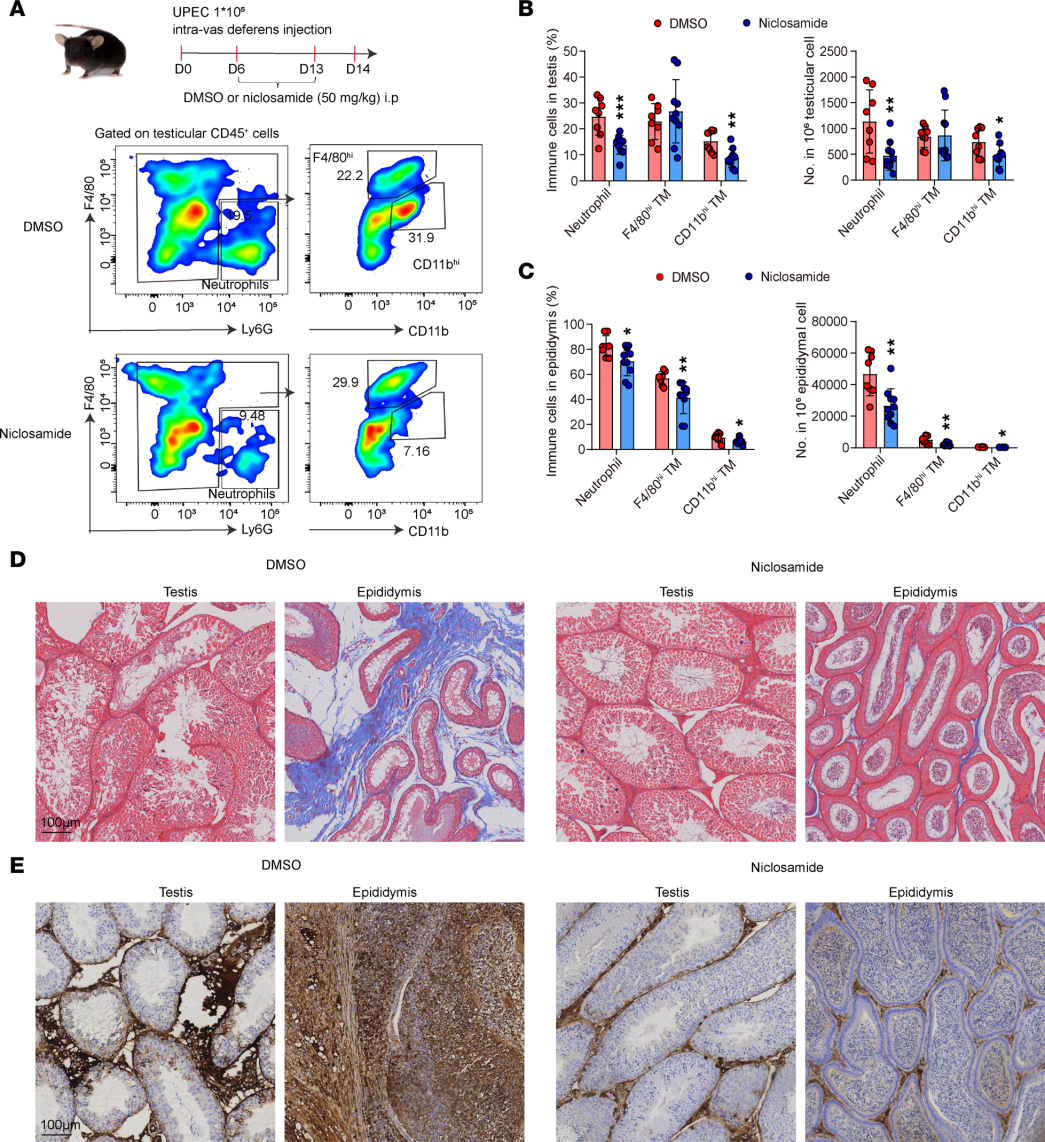
Niclosamide inhibits tissue damage and fibrosis in UPEC-induced epididymo-orchitis. (**A**–**C**) Mice were treated with niclosamide (50 mg/kg) from day 6 to day 13 via peritoneal injection. Flow cytometry was used to quantify immune cell populations in the testis and epididymis, and representative images and bar plots are shown. DMSO: *n* = 8; niclosamide: *n* = 10. Data are presented as the mean ± SD. *P < 0.05, **P < 0.01, and ***P < 0.001, by Student’s *t* test. (**D** and **E**) Representative Masson’s staining and collagen I staining were used to visualize tissue structure and fibrosis in the testis and epididymis. Scale bar: 100 μm.

Having demonstrated that S100a4^+^ TMs contribute to fibrosis of the testes and epididymis after UPEC infection, we next investigated the therapeutic effect of inhibiting S100a4^+^ macrophage function. To do this, we treated mice with the S100a4 inhibitor niclosamide and observed the impact on fibrotic pathology in the UPEC-induced epididymo-orchitis model. Our analyses revealed that niclosamide treatment significantly reduced the numbers and percentages of immune cells detected in both the testis and epididymis (Figure 7, A–C). Masson and collagen I immunohistochemical staining confirmed that niclosamide treatment effectively prevented fibrosis of these tissues (Figure 7, D and E). Additionally, niclosamide treatment significantly inhibited the accumulation of S100a4^+^CD11b^+^ macrophages in both the testis and epididymis (Supplemental Figure 6, A and B). These results further demonstrate that inhibition of S100a4 is an effective strategy for reducing fibrosis in UPEC-induced epididymo-orchitis.

## Discussion

TMs are a diverse population that plays critical roles in tissue development and homeostasis (6, 10), but the subtypes that drive inflammation and fibrosis in response to infection remain unclear. In this study, we demonstrate that UPEC-induced orchitis substantially remodels the murine TM compartment, with marked accumulation of S100a4^+^ monocyte-derived macrophages in the testis and epididymis, as well as a corresponding enrichment of this population in fibrotic areas of human surgical specimens. These macrophages differentiate locally and adopt a myofibroblast state, characterized by production of large quantities of collagen I and fibronectin. Importantly, selective targeting of S100a4^+^ macrophages using either genetic tools or pharmacological approaches effectively inhibited the progression of epididymal and testicular fibrosis in vivo. Our findings provide compelling evidence that transition of S100a4^+^ macrophages toward a myofibroblast state under the control of TGF-β/STAT3 plays a crucial role in UPEC infection–induced epididymal and testicular fibrosis.

In a previous study, we identified 2 distinct populations of TMs in murine testis and epididymis (1), but the key functions of these subsets during tissue inflammation remained unexplored. Moreover, the testis and epididymis exhibit distinct tubular architectures and immunological profiles. Building on our prior demonstration that distal epididymis regions (corpus and cauda), unlike proximal regions (initial segment and caput), develop a proinflammatory neutrophil/monocyte niche after UPEC infection (24), we now find testicular tissue similarly develops substantial neutrophil/monocyte enrichment after UPEC challenge, thereby recapitulating the immune response pattern of the corpus/cauda epididymis. In the present study, we used scRNA-Seq approaches to uncover a notable increase in interstitial macrophage–like macrophages expressing *Cd72* and *Aoah* at day 7 after UPEC infection, suggesting local differentiation of this subset within the inflamed tissue. In parallel, we observed a marked decrease in the percentage of the peritubular macrophage–like resident population that mediates spermatogonial stem cell development (25). Previous studies have also shown that UPEC infection stimulates accumulation of both F4/80^hi^ tissue-resident macrophages and CD11b^hi^ monocyte-derived macrophages in the testis and epididymis (1, 11). Here, we observed that a third population of TMs also emerges during acute UPEC infection, characterized by high expression of monocyte marker *Ccr2* together with fibrosis-associated marker *S100a4*, which was associated with ECM deposition and tissue damage in both mouse orchitis and human pathological specimens.

Testicular fibrosis is a pathological condition marked by excessive accumulation of ECM proteins, including collagens (13, 14). This leads to scarring and hardening of the testicular tissue, thereby disrupting normal structure and function to ultimately impair male fertility (10, 26). Testicular fibrosis can arise from multiple factors, including infection, trauma, autoimmune reactions, and systemic disease (3), but is most often linked with chronic inflammation leading to activation of fibroblasts (14). Previous studies have highlighted a critical role of the inflammatory immune microenvironment in testicular fibrosis, particularly for newly recruited CCR2^+^ macrophages (10, 13). Consistent with these findings, we identified a S100a4^+^CCR2^+^ monocyte–derived TM subset that contributes to fibrosis in the testis and epididymis during UPEC-induced epididymo-orchitis. These CCR2^+^ macrophages were capable of producing substantial amounts of ECM components, including fibronectin, collagen I, and MMPs, as well as PDGFs, all of which are pivotal in fibrosis development (13). Indeed, earlier studies have shown that CCR2-KO mice exhibit a marked reduction in immune cell infiltration, fibrosis severity, and tissue damage in this model, suggesting that targeting CCR2^+^ monocyte-macrophage infiltration could be a potential therapeutic strategy for UPEC-orchitis (1, 13). Our data further reveal that these cells can adopt a myofibroblast state and directly contribute to the fibrotic process in UPEC-induced epididymo-orchitis in vivo, whereas therapeutic targeting of S100a4^+^ TM substantially reduced pathology.

S100a4 has emerged as a key factor in the development of fibrosis by influencing a range of processes, including cell motility, invasion, and ECM remodeling (27, 28). Intracellularly, S100a4 interacts with nonmuscle myosin 2, which is crucial for myofibroblast transdifferentiation and contractile functions (28, 29). Extracellularly, S100a4 triggers inflammatory responses that lead to fibrogenesis (30). In our study, we observed that S100a4^+^ macrophages and S100a4-stimulated BMDMs exhibit increased collagen I production. Our findings suggest S100a4^+^ monocytes mediate testicular fibrosis progression through 2 synergistic mechanisms: (a) paracrine activation of neighboring monocytes/macrophages via extracellular S100a4 secretion, stimulating excessive ECM deposition; and (b) autonomous differentiation into a myofibroblast-like phenotype. These complementary pathways collectively amplify fibrotic remodeling after UPEC infection. Notably, knocking out S100a4 in Lyz2^+^ BMDMs reduced fibrosis and tissue damage in the testis and epididymis. These findings suggest targeting both the intracellular and extracellular functions of S100a4 could be potential interventions to mitigate fibrosis and tissue injury in bacterial infections of the reproductive organs.

The TGF-β/SMAD3 signaling pathway is integral to the pathogenesis of fibrotic diseases (21, 31). Previous research indicates S100a4 binds to SMAD3, enhances transcriptional activity of the SMAD3/SMAD4 complex (32), and thereby promotes TGF-β–induced activation of fibroblasts. Additionally, TGF-β can directly activate the JAK-STAT3 axis, which facilitates hepatic fibrosis in conjunction with the SMAD pathway (33). This coordinated action results in upregulation of fibrosis-associated proteins such as α-SMA, collagen-1, and fibronectin (32). Notably, inhibiting SMAD phosphorylation effectively impedes S100a4-mediated transdifferentiation of fibroblasts into myofibroblasts and, therefore, may represent a therapeutic target for multiple fibrotic conditions (34). Indeed, our current findings demonstrate S100a4 can activate the p-STAT3 pathway and induce MMT, potentially in a SMAD-independent manner. Inhibiting p-STAT3 could, therefore, be a strategy for reducing mRNA levels of ECM proteins in the treatment of fibrotic diseases. TLR4 has been previously characterized as a receptor mediating S100A4 signaling; our study reveals a distinct pathological mechanism underlying UPEC-induced epididymo-orchitis. In contrast to the canonical TLR4/MyD88-driven inflammation-fibrosis axis observed in epididymal models (2), we demonstrate that testicular MMT is orchestrated through a S100a4-STAT3-SMAD3 signaling cascade, highlighting tissue-specific regulatory divergence in inflammatory fibrogenesis.

Recent studies have reported that S100a4 may promote the progression of LPS-induced epididymitis and contribute to reduced sperm vitality (35). Additionally, S100a4 has been shown to increase the susceptibility of a macrophage subpopulation to Zika virus infection infection, thus facilitating virus invasion and persistence within the seminiferous tubules of testis (36). S100a4^+^ monocytes/macrophages also have been implicated in sustaining long-term Zika virus infection in the testes (37). Despite these findings, the function of S100a4 in response to bacterial infections in the testis was not completely understood.

Our study highlights the underappreciated role of S100a4 during UPEC infection in modulating macrophage phenotype to promote fibrosis. Inhibition of S100a4 with niclosamide or macrophage-specific S100a4 KO substantially reduced immune cell infiltration, tissue damage, and fibrosis in infected murine models. Our findings establish the critical role of S100a4^+^ macrophages in fibrosis during UPEC-induced epididymo-orchitis and propose them as potential targets for antifibrotic therapy development. Additional functions of S100a4^+^ macrophages in this context are also possible, including modulation of monocyte infiltration and/or promote bacterial clearance, hence further investigation is clearly warranted.

## Methods

### Sex as a biological variable.

Because this study is about the male reproductive system, only testicular and epididymal tissues from men with epididymo-orchitis were included. And only male mice were used in this study.

### Animals.

Male WT C57BL/6J mice were acquired from Charles River Laboratories in Beijing, China. Our laboratory also houses various transgenic strains: generation of the S100a4-EGFP mice (S100a4-GFP; strain 012893) was described in a previous report (38). C57BL/6-background S100a4-floxed mice (strain T017549) were generated by GemPharmatech. Macrophage-specific S100a4-KO (*S100a4^fl/fl^ Lyz2^Cre^*) was generated by crossing S100a4-floxed mice with lysozyme M (Lyz2)-Cre mice (strain no. T003822).

### Human tissue biopsies.

Bouin’s solution–fixed, paraffin-embedded sections of testicular and epididymal tissue were supplied by the Department of Urology at the First Affiliated Hospital of Zhengzhou University. Informed consent was obtained and detailed patient information is provided in Supplemental Table 1.

### UPEC-induced epididymo-orchitis model.

The UPEC-induced epididymo-orchitis mouse model was established using a previously described protocol (11). Adult male mice, aged 10–12 weeks, received an injection of 1 × 10^5^ UPEC (strain 536) suspended in 10 μL of 0.9% saline and administered via the vas deferens, using a 30G syringe. Sham-operated control mice received a 10 μL saline injection. To prevent UPEC leakage, the urethral side of the vas deferens was ligated prior to injection. The mice were then sacrificed either on day 7 or day 14 after injection before the testes and epididymis were collected for analysis.

### BMT.

To delineate the developmental origin of testicular S100a4^+^ TMs, BMT was performed using S100a4-GFP donor mice. WT recipient mice (8-week-old male mice) underwent myeloablation via 7.5 Gy whole-body x-ray irradiation (X-RAD 320, Precision X-Ray). After 24-hour recovery in specific pathogen-free conditions, 5 × 10^6^ BM cells isolated from S100a4-GFP donors were intravenously engrafted via tail vein injection. After a 4-week engraftment period, the UPEC-induced epididymo-orchitis mouse model was established. Testicular and epididymal tissues were harvested at postinfection day 7.

### scRNA-Seq.

For scRNA-Seq, testes from UPEC-infected mice were harvested on days 7 and 14 (or on day 7 for the sham-operated controls). The testes were then dissociated into a single-cell suspension by incubation in digestion buffer (RPMI-1640 medium, 10% FBS, 1 mg/mL collagenase I, and 1 unit/mL DNAse I) at 37°C for 30 minutes. The cells were subsequently filtered through a 70 μm strainer then stained with phycoerythrin anti-mouse CD45 antibody (103106, BioLegend) for 15 minutes at 4°C. The enriched CD45^+^ immune cells were then isolated using a BD Biosciences flow cytometer (BD FACSAria III). After sorting, a Dead Cell Removal Kit (17899, STEMCELL Technologies) was used to eliminate any nonviable CD45^+^ immune cells prior to sequencing. Next, 10^4^ viable cells, gel beads containing cell barcodes, and reaction reagents were encapsulated in oil droplets to create gel beads in emulsion (GEMs) using the DG1000 system (BMKMANU, Biomarker Technologies). RNA from individual cells was then released into the GEMs. In a controlled environment, RNA molecules were annealed with poly(dT) primers that included a cell barcode and Unique Molecular Identifier, which initiated synthesis of the complementary DNA strand. This was followed by the addition of a cytosine nucleotide triplet at the end of the newly formed strand. Reverse transcription was completed using template switching oligos, which hybridize with the cytosine-rich overhang via their rGrGrG sequence. The GEMs were subsequently broken down, allowing for isolation and PCR amplification of the cDNA (which was quantified using the Qubit 4.0 Fluorometer and integrity assessed using the Agilent 2100 Bioanalyzer). After rigorous quality control, a next-generation sequencing platform was used for sequencing. The FASTQ files were processed using BSCMATRIX to generate single-cell feature-barcoded matrices.

### scRNA-Seq data processing and analysis.

The Biomarker Technologies Corporation processed the scRNA-Seq data and performed bioinformatic analysis. The data matrices were loaded in R (version 4.4). A Seurat object was created using the following filtering parameters: nFeature-RNA <7,000; nFeature-RNA >500; and percent.mt <20. Next, *t*-distributed stochastic neighbor embedding and uniform manifold approximation and projection methods were used for cell clustering. The “FindAllMarkers” function was used to identify the conserved marker genes in clusters using default parameters.

### Flow cytometry.

To identify immune populations, testes and epididymis were disaggregated into cell suspensions for flow cytometry analysis. Initially, 10^6^ cells were incubated with anti-CD16/32 antibody for 10 minutes to block nonspecific immunoglobulin binding to Fc receptors. Next, the cells were stained using combinations of antibodies for 30 minutes at 4°C, then washed and resuspended in FACS buffer (1% BSA in PBS). The immune cell populations were then analyzed using a BD Biosciences flow cytometer (FACSAria III), and the resulting data were processed using FlowJo software, version 10. The antibodies are listed in Supplemental Table 2.

### Histological staining.

To assess tissue damage and immune cell infiltration, the testes and epididymis were harvested, fixed in Bouin’s solution for 4 hours, and subsequently embedded in paraffin. Tissue sections of 6 μm thickness were then stained; H&E staining was used for the testes, and Masson’s trichrome stain was applied for connective tissue visualization. Imaging was performed using a scanning microscope (Panoramic MIDI).

### Multiplex IF staining.

We performed multiplex IF staining using the TSAPLus staining kit (Servicebio Technology) following the manufacturer’s instructions. Initially, we deparaffinized the 6 μm paraffin slides and rehydrated them using a dewaxing solution and pure ethanol. We then subjected the slides to antigen repair with citric acid (pH 6.0) and blocked with 3% BSA for 30 minutes. The slides were incubated with primary antibody at 4°C overnight, followed by incubation with the corresponding HRP-labeled secondary antibody at room temperature (RT) for 1 hour. Next, we added the corresponding tyramide signal amplification (TSA) and incubated the slides at RT for 10 minutes. After this, we placed the slides back in citric acid antigen repair buffer and boiled them for 10 minutes in a microwave oven. The sections were then blocked again using 10% rabbit serum before adding the second primary antibody, followed by the corresponding HRP and a different fluorescence-labeled TSA. This process was repeated for each primary antibody used. Finally, nuclei were stained with DAPI and slides were sealed using anti–fluorescence-quenching tablets. Images were acquired using digital slide scanners (Panoramic MIDI). Primary antibodies are listed in Supplemental Table 3.

### BMDM isolation.

BMDMs were generated from the BM of WT or transgenic mice according to published protocols (11). BMDMs were cultured in complete RPMI-1640 medium supplemented with 10% FBS (Gibco, Thermo Fisher Scientific), 100 IU/mL penicillin, and 100 μg/mL streptomycin (Gibco, Thermo Fisher Scientific). Cultures were maintained at 37°C in a 5% CO_2_ incubator. Macrophage CSF (M-CSF) (20 ng/mL) was added to induce BMDM polarization. The BMDMs were cultured for 7 days and stimulated with either S100a4 (100 ng/mL), TGF-β (5 ng/mL), niclosamide (10 μM), or a combination of these for the indicated times.

### Western blot.

Primary BMDMs were lysed using RIPA buffer supplemented with a cocktail of phosphatase and protease inhibitors. Protein concentration of the lysates was determined using a bicinchoninic acid assay kit (PC0020; Solarbio). A total of 20 μg of protein was then separated by SDS-PAGE and subsequently transferred electrophoretically onto nitrocellulose filter membranes (HATF00010, MilliporeSigma). The membranes were blocked with 5% nonfat milk before overnight incubation at 4°C with primary antibodies. Then the membranes were washed and incubated with the appropriate secondary antibodies at RT for 1 hour. Protein bands were visualized using an enhanced chemiluminescence reagent in the ChemiDoc MP imaging system (Bio-Rad). Primary antibodies are listed in Supplemental Table 4.

### qRT-PCR.

Total RNA was isolated from BMDMs using Trizol reagent and then reverse transcribed into cDNA using PrimeScript RT Master Mix. The quantity of mRNA was measured with TB GreenPremix Ex Taq II reagent (RR820A, Takara) and detection was carried out on an Agilent Mx3005P qPCR instrument, following the manufacturer’s instructions. Relative mRNA expression levels across different groups were calculated using the comparative Ct method, with β-actin serving as the internal control. Primer sequences are detailed in Supplemental Table 5.

### RNA-Seq.

To identify differentially expressed genes in S100a4 KO macrophages, BMDMs were first isolated from *S100a4^fl^/^fl^* or *S100a4^fl/fl^ Lyz2^Cre^* mice and then cultured with M-CSF (20 ng/mL) for 7 days. RNA extraction was performed using the RNeasy mini kit (QIAGEN). Sequencing of the extracted RNA was carried out on an Illumina HiSeq 2500 system, provided by Shanghai OE Biotech. Quality checks of the sequence reads were conducted using FastQC. Data analysis was performed with EdgeR software, setting the threshold for significantly differentially expressed genes at a *P* value of less than 0.05 and a fold change of greater than 1.5. The resulting RNA-Seq data have been made publicly available in the Gene Expression Omnibus (GEO) database under accession number GSE281537.

### Statistics.

Statistical analyses were performed using an unpaired Student’s *t* test when comparing 2 groups, or 1-way ANOVA followed by Tukey’s post hoc test for comparisons involving more than 2 groups. A *P* value of less than 0.05 was considered statistically significant.

### Study approval.

All mice were bred and maintained under specific pathogen-free conditions within the animal facilities of Zhengzhou University. Ethical conduct of all animal experiments was reviewed and approved by the Ethics Committee of Zhengzhou University (approval no. KY154). Human epididymal and testicular specimens were procured during routine open surgery after study approval by the Ethics Committee of Zhengzhou University (approval no. KY154), ensuring compliance with the standards outlined in the Declaration of Helsinki.

### Data availability.

The RNA-Seq data were uploaded in the GEO database with the accession code GSE281537. The scRNA-Seq data were uploaded in the GEO database with the accession code PRJNA1189862. Values for all data points in graphs are reported in the Supporting Data Values file.

## Author contributions

MW, SB, and ZZ conceptualized the study and acquired funding for the work. MW, XC, ZF, LC, PW, HW, ZW, YZ, YD, and ZZ contributed to the study methodology. MW, XC, and SB conducted the computational data analysis. MW supervised the study. MW and ZZ wrote the original draft of the manuscript. MW, XC, ZF, LC, HW, PW, SB, and ZZ reviewed and edited the manuscript.

## Supplementary Material

Supplemental data

Unedited blot and gel images

Supporting data values

## Version 1 10/01/2025

Electronic publication

## Version 2 07/30/2026

Revised supplemental figure 4 legend and supporting data values for figure 5B
