# Supplementary material for: Macrophage transition to a myofibroblast state drives fibrotic disease in uropathogenic E. coli-induced epididymo-orchitis
Source: J Clin Invest. 2025 Oct 1;135(19):e193793. doi: 10.1172/JCI193793 (PMC12483606; doi:10.1172/JCI193793)
Supplement: Supplemental data [file jci-135-193793-s008.pdf]

Supplemental Figures

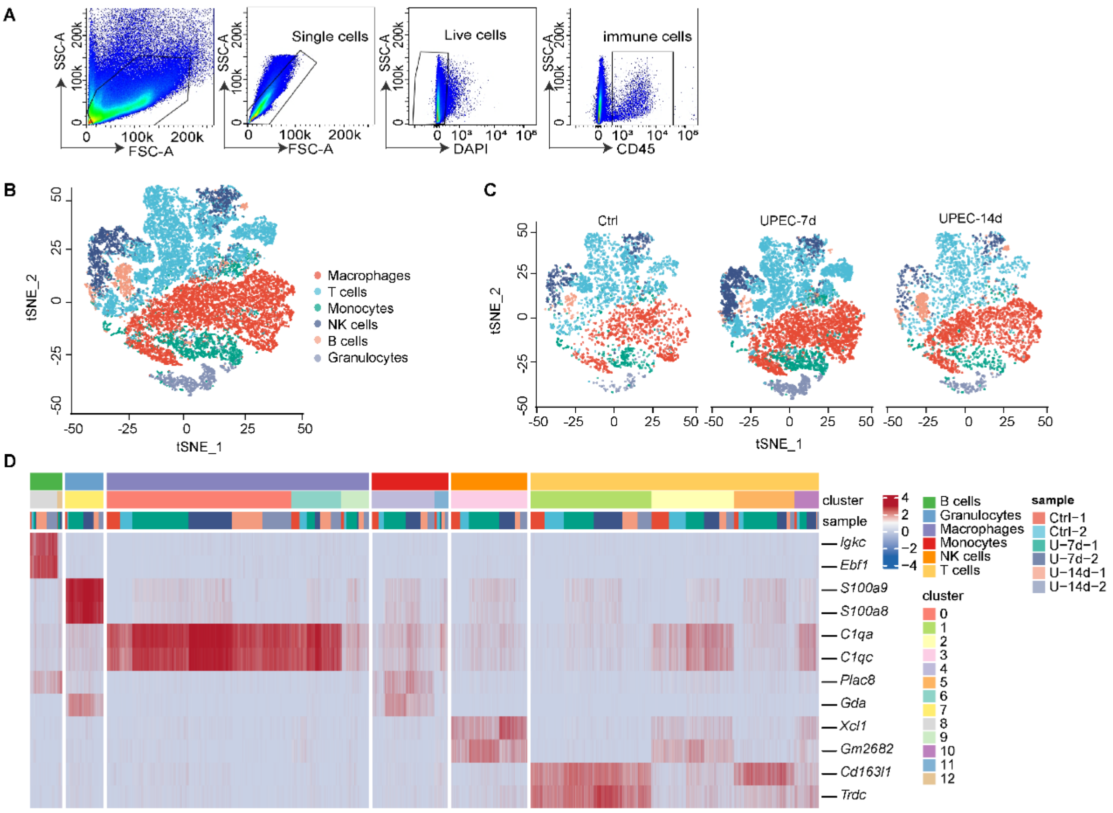

Supplemental Figure 1 UPEC infection modifies the immune compartment of mouse testes. (A) FACS gating strategy. (B-C) t-SNE dimensionality reduction clustering analysis of immune cell subpopulations in the testes following UPEC infection, shown in summary and by different groups. (D) Bar graphs illustrating changes in the number and proportion of immune cells in the testes at various time points after UPEC infection.

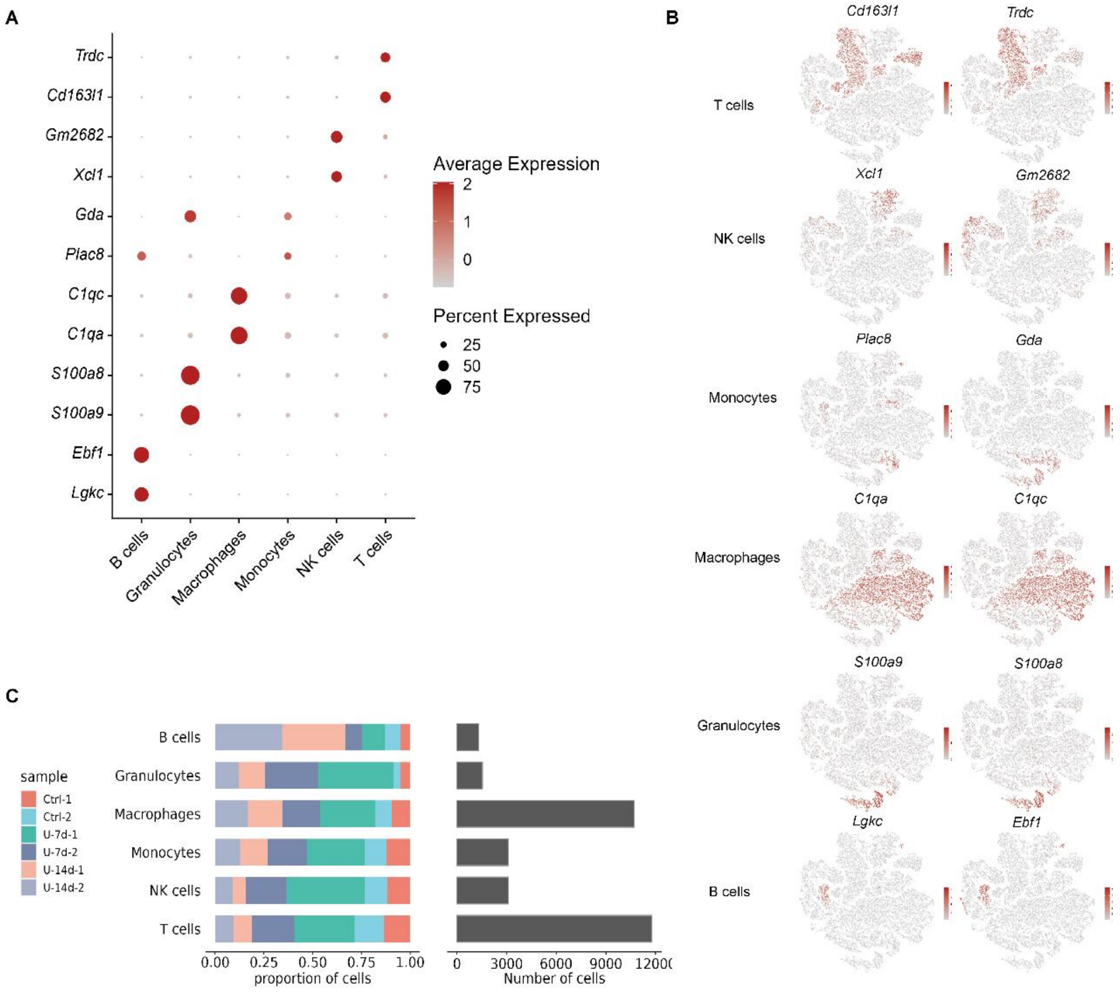

Supplemental Figure 2 The number and proportion of testicular immune cells were changed following UPEC infection. (A-B) Top 2 immune cell markers in each cluster, showing both dot plots and tSNE plots. (C) Bar graphs illustrating changes in the number and proportion of immune cells in the testes at various time points after UPEC infection.

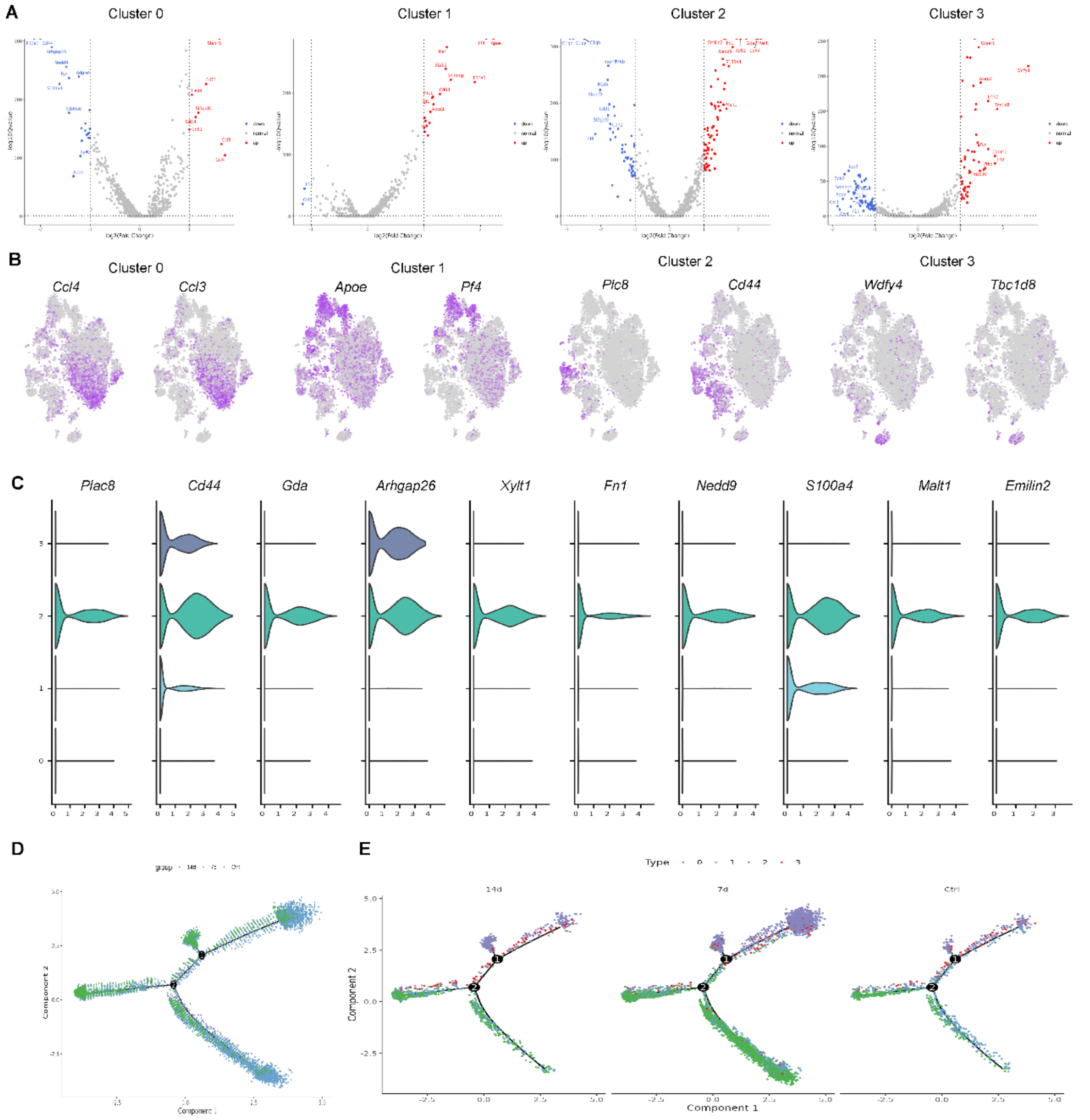

Supplemental Figure 3 UPEC infection remodels the TM compartment during different stages of inflammation. (A) Heat-map showing DEGs that define Cluster 0, 1, 2 and 3 TM. (B) tSNE plots visualizing the top two macrophage markers per TM cluster. (C) Violin plot showing the top 10 marker genes in cluster 2 macrophages. (D) Monocle prediction of macrophage developmental trajectory. (E) Monocle prediction of TM developmental trajectory with each timepoint shown separately.

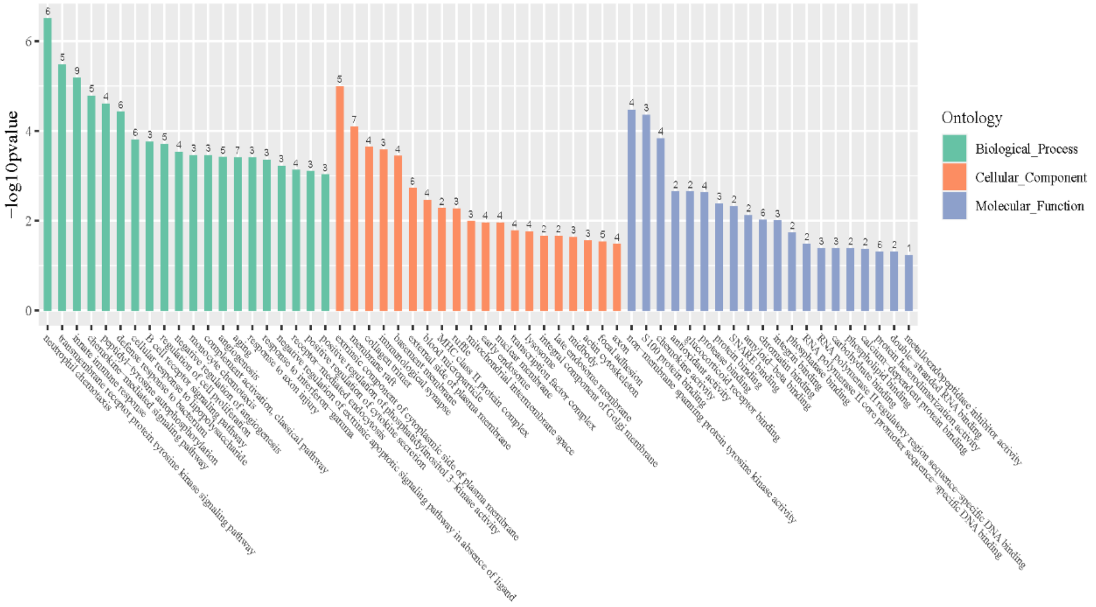

Supplemental Figure 4 GO (Gene Ontology) analysis summarizing signaling pathways that were highly enriched in Cluster 2 macrophages.

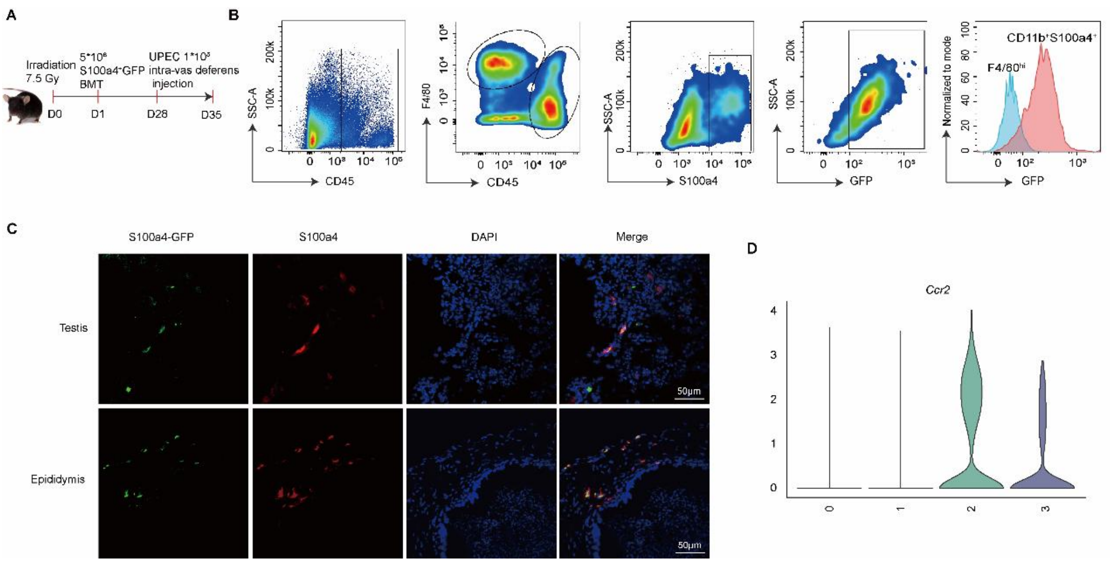

Supplemental Figure 5 Monocyte-Derived S100a4⁺ Tissue-Resident Macrophages in UPEC-Induced Epididymo-Orchitis. (A) Schematic workflow of BMT and subsequent UPEC-induced epididymo-orchitis model. (B) Flow cytometric identification of S100a4-GFP⁺ cells within CD11b⁺S100a4⁺ TM and F4/80^{hi} TM. (C) Immunofluorescence co-localization analysis of S100a4 (red) and S100a4-GFP⁺ cells (green) in testicular and epididymal sections. Nuclei counterstained with DAPI (blue). Scale bars: 100 μm. (D) Violin plot quantifying *Ccr2* expression across TRM clusters (Cluster 0-3) from scRNA-seq data.

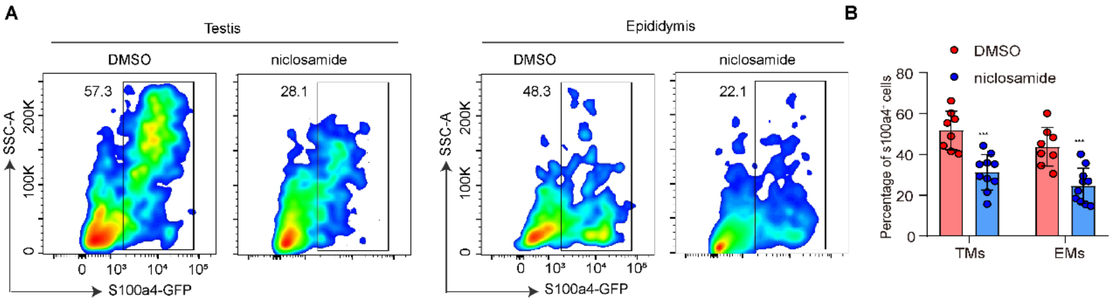

Supplemental Figure 6 Niclosamide inhibited the accumulation of S100a4^{+} cells in testis and epididymis. (A-B) Images and bar plots showing the percentage of S100a4^{+} cells among CD45^{+}F4/80^{+}CD11b^{+} macrophages in testis and epididymis after niclosamide treatment. DMSO: n = 8, niclosamide: n = 10; mean ± SD, Student's *t* test , ***p < 0.001.

Supplemental Table 1：Patient information.

Patient	Age	Treatment	Pathology diagnosis
1	67	Orchiectomy (right)	Chronic inflammation with hyperplasia of granulation tissue
2	54	Orchiectomy (right)	Chronic inflammation infiltration
3	61	Orchiectomy (left)	Chronic inflammation infiltration with hyperplasia of granulation tissue
4	48	Orchiectomy (left)	Chronic inflammation infiltration with superficial necrosis
5	65	Orchiectomy (left)	Chronic inflammation infiltration with fibrotic nodules
6	71	Orchiectomy (left)	Chronic inflammation with fibrotic nodules
7	74	Epididymectomy (right)	Chronic inflammation infiltration
8	32	Epididymectomy (right)	Chronic inflammation infiltration
9	68	Epididymectomy (right)	Chronic inflammation infiltration with fibrotic nodules
10	75	Epididymectomy (left)	Chronic supportive inflammation with superficial necrosis
11	57	Epididymectomy (left)	chronic epididymitis with acute immune response and spermatic granuloma
12	61	Epididymectomy (left)	Chronic inflammation infiltration

Supplemental Table 2：Antibodies used for flow cytometry.

Antibody Name	Dilution	Catalog	Supplier	Identifier
Alexa Fluor® 700 anti-mouse CD45 antibody	1:100	103128	BioLegend	RRID:AB_493715
APC/Cy7 anti-mouse/Human CD11b antibody	1:100	101226	BioLegend	RRID:AB_830642
PE/Cy7 anti-mouse F4/80 antibody	1:100	123114	BioLegend	RRID:AB_893478
Brilliant violet 510 anti-mouse Ly-6G/Ly-6c(Gr-1) antibody	1:100	108438	BioLegend	RRID:AB_2562215
FITC anti-mouse CD16/32 antibody	1:100	101301	BioLegend	RRID:AB_312800
S100a4 polyclonal antibody	1:100	16105-1-AP	Proteintech	RRID:AB_11042591
DAPI	1:500	AR1776	BOSTER	

Supplemental Table 3：Antibodies used for immunofluorescence.

Antibody Name	Dilution	Catalog	Supplier	Identifier
F4/80	1：200	#30325	Cell Signaling Technology	RRID:AB_2798990
CD11b	1：200	#AB62817	Abcam	RRID:AB_955740
Collagen I	1：200	#72026	Cell Signaling Technology	RRID:AB_2904565
α-SMA	1：200	#AB5694	ABcam	RRID:AB_2223021
S100a4	1:100	16105-1-AP	Proteintech	RRID:AB_11042591

Supplemental Table 4: Primary antibodies used for Western blot.

Antibody Name	Dilution	Catalog	Supplier	Identifier
SMAD2	1:1000	#5339	Cell Signaling Technology	RRID:AB_10626777
Phospho-SMAD2	1:1000	#3108	Cell Signaling Technology	RRID:AB_490941
Smad3	1:1000	#9523	Cell Signaling Technology	RRID:AB_2193182
Phospho-Smad3	1:1000	#9520	Cell Signaling Technology	RRID:AB_2193207
AKT	1:1000	#9272	Cell Signaling Technology	RRID:AB_329827
Phospho-Akt	1:1000	#4060	Cell Signaling Technology	RRID:AB_2315049
p38MAPK	1:1000	#8690	Cell Signaling Technology	RRID:AB_10999090
Phospho-p38MAPK	1:1000	#4511	Cell Signaling Technology	RRID:AB_2139682
ERK1/2	1:1000	#A16686	ABclonal	RRID:AB_2770274
Phospho-ERK1/2	1:1000	#AP0974	ABclonal	RRID:AB_2863871
STAT3	1:1000	#A19566	ABclonal	RRID:AB_2862671
Phospho-STAT3	1:1000	#AP0705	ABclonal	RRID:AB_2863810
β-Actin	1:8000	#AC006	ABclonal	RRID:AB_2768236
Collagen I	1:1000	#AB34710	ABcam	RRID:AB_731684
α-SMA	1:1000	#AB5694	ABcam	RRID:AB_2223021
Fibronectin	1:1000	#15613-1-AP	proteintech	RRID:AB_2105691

Supplemental Table 5: Primer sequences used for RT-qPCR.

Gene	Forward Primer	Reverse Primer
Tnfa	GGTGCCTATGTCTCAGCCTCTT	GCCATAGAACTGATGAGAGGGAG
Il6	TACCACTTCACAAGTCGGAGGC	CTGCAAGTGCATCATCGTTGTTC
Il-1b	TGGACCTTCCAGGATGAGGACA	GTTCATCTCGGAGCCTGTAGTG
Il-1a	ACGGCTGAGTTTCAGTGAGACC	CACTCTGGTAGGTGTAAGGTGC
Il10	CGGGAAGACAATAACTGCACCC	CGGTTAGCAGTATGTTGTCCAGC
Ccr2	GCTGTGTTTGCCTCTCTACCAG	CAAGTAGAGGCAGGATCAGGCT
Ccl2	GCTACAAGAGGATCACCAGCAG	GTCTGGACCCATTCCTTCTTGG
Col1a1	CCTCAGGGTATTGCTGGACAAC	CAGAAGGACCTTGTTTGCCAGG
Col1a2	TTCTGTGGGTCCTGCTGGGAAA	TTGTCACCTCGGATGCCTTGAG
Mmp9	GCTGACTACGATAAGGACGGCA	TAGTGGTGCAGGCAGAGTAGGA
Mmp11	GATTGATGCTGCCTTCCAGGATG	CAGCGGAAAGTATTGGCAGGCT
Acta2	TGCTGACAGAGGCACCACTGAA	CAGTTGTACGTCCAGAGGCATAG
Fn1	CCCTATCTCTGATACCGTTGTCC	TGCCGCAACTACTGTGATTCGG
Tert	GAAAGTAGAGGATTGCCACTGGC	CGTATGTGTCCATCAGCCAGAAC
Thbs1	GGTAGCTGGAAATGTGGTGCGT	GCACCGATGTTCTCCGTTGTGA
Gapdh	TCTCTGCTCCTCCCTGTTCC	TACGGCCAAATCCGTTCACA
