# Supplementary figures and images for: Macrophage transition to a myofibroblast state drives fibrotic disease in uropathogenic E. coli-induced epididymo-orchitis
Source: J Clin Invest. 2025 Oct 1;135(19):e193793. doi: 10.1172/JCI193793 (PMC12483606; doi:10.1172/JCI193793)

Full unedited gel for Figure 4E

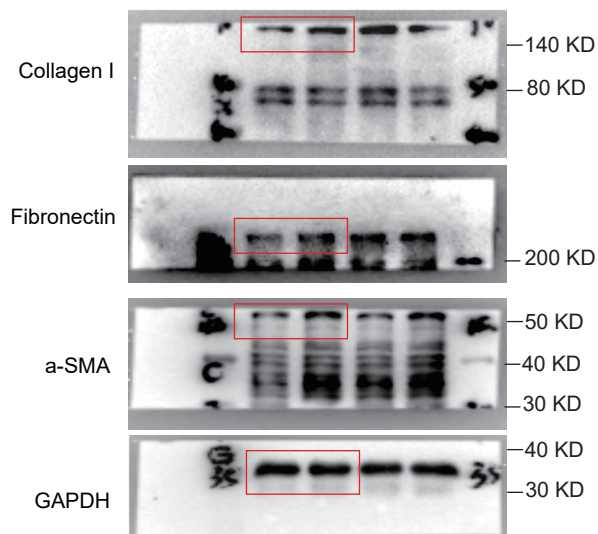

Full unedited gel for Figure 5A

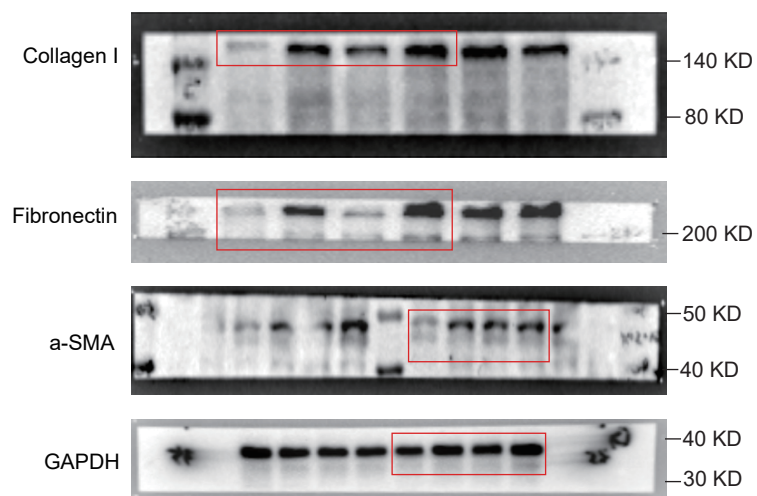

Full unedited gel for Figure 5D

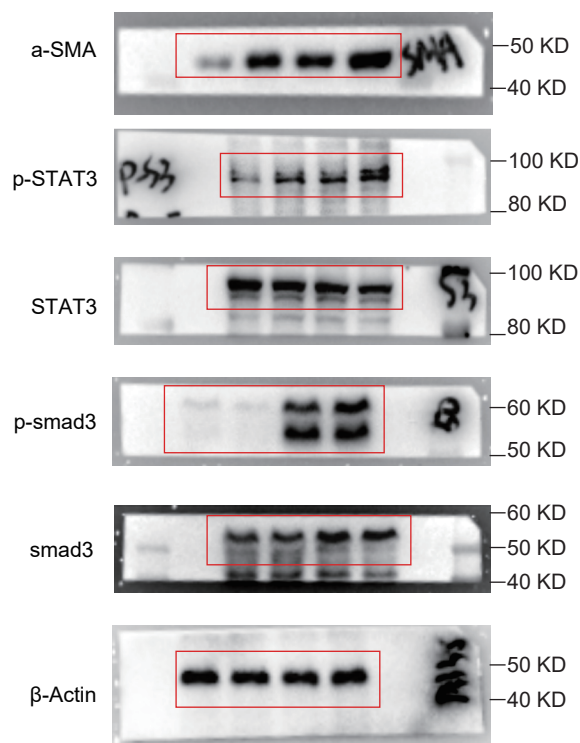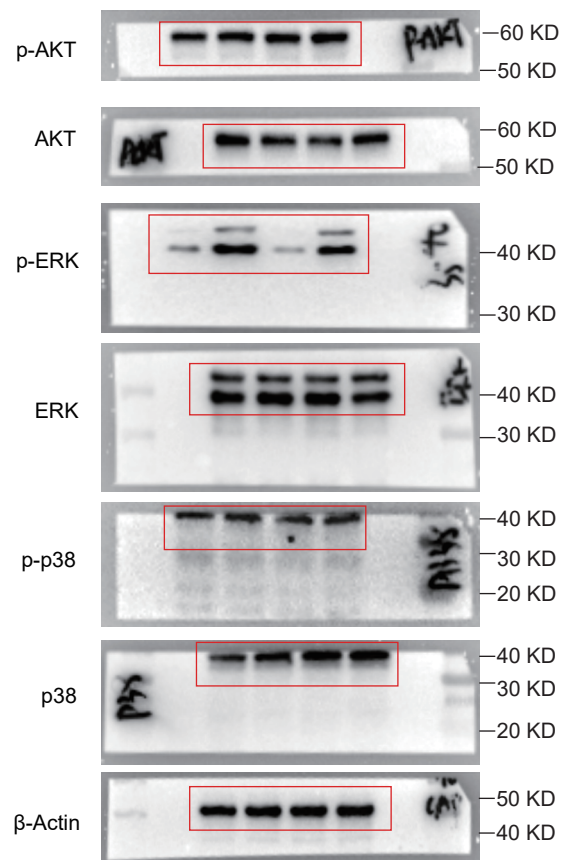

Supplement: Unedited blot and gel images [file jci-135-193793-s009.pdf]
